# Restricting the induction of NGF in ovarian stroma engenders selective follicular activation through the mTOR signaling pathway

**DOI:** 10.1038/cddis.2017.168

**Published:** 2017-05-25

**Authors:** Yuanlin He, Xiaoxu Peng, Tinghe Wu, Weijie Yang, Wenwen Liu, Jing Zhang, Yiping Su, Feifei Kong, Xiaowei Dou, Jing Li

**Affiliations:** 1 State Key Laboratory of Reproductive Medicine, Nanjing Medical University, Nanjing, China; 2 Department of Biotechnology and Biomedicine, Yangtze Delta Region Institutes of Tsinghua University, Jiaxing, China; 3 Department of Gynecology, Obstetrics and Gynecology Hospital Affiliated to Nanjing Medical University, Nanjing, China

## Abstract

In mammalian ovaries, primordial follicles remain in a quiescent state until activation by the surrounding microenvironment. Ovarian intervention, for example, ovarian cystectomy, ovarian wedge resection or laser drilling therapies for polycystic ovarian syndrome, has long been reported to change follicular development by an unknown mechanism(s). Herein, we established a murine model with partial ovarian resection of one ovary unilaterally, with the contralateral ovary undamaged. We found the injury accelerated follicular activation and development through the mTORC1 signaling pathway. Moreover, the stimulation of primordial follicles was restricted near the incision site where the mTORC1 pathway showed sequential activation beginning at the interstitial cells and proceeding to the primordial follicles. Total and polysome-associated RNA-seq revealed the increase of the nerve growth factor (NGF) family member, in both two fractions and immunostaining showed the restricted induction of NGF near the incision site. In cultured newborn ovaries, NGF demonstrated increase of follicular activation, and moreover, the NGF inhibitor K252a effectively blocked activation of primordial follicles stimulated by the surgery. We liken ovulation in mammals to minor tissue trauma, which happens naturally and cyclically in the body. As the increase in NGF accompanied the accumulation of activated primordial follicles after ovulation, our study may represent a common mechanism for selective follicular activation induced by a localized increase in NGF in interstitial cells and mediated via the mTOR signaling pathway. In addition, the NGF inhibitor K252a and the mTOR inhibitor rapamycin constitute good candidates for protecting follicular reserve against over exhaustion after ovarian surgery.

In mammals, it is widely accepted that primordial follicles are assembled in finite numbers in the ovary before or around birth and remain quiescent, sometimes for decades.^1^ Throughout the reproductive years, the number of primordial follicles is gradually depleted by continual recruitments until the pool is exhausted at menopause.^2^ Therefore, the recruitment rate of primordial follicles is one determinant of how long the primordial follicle pool will last. In humans, one of the most frequent causes of sterility is premature ovarian insufficiency (POI), which affects approximately 1–2% of women under 40 years of age, and is characterized by the disappearance of menstrual cycles and associated with early depletion of primordial follicles.^3^

Activation of primordial follicles involves the transition from primordial follicle to primary follicle, entailing oocyte growth, granulosa cell differentiation from flat to cuboidal and theca cell recruitment.^4^ By using transgenic mouse models, a key pathway—the intra-oocyte PI3K/mTOR pathway—is emerging as crucial during the transition from primordial-to-primary follicles. Indeed, premature depletion of primordial follicles is seen in knockout mice lacking genes of this pathway, such as *Pten*, *Akt1*, *Foxo3*, *rpS6* or *Tsc1/Tsc2*.^5, 6, 7^ However, because this pathway can be activated by various hormones, growth factors and cytokines, how the upstream extra-oocyte signals govern the activation of the intra-oocyte signaling pathway remains poorly understood. A recent study by Liu *et al.* provides a picture of how mammalian primordial follicles are activated; that is, the microenvironment surrounding primordial follicles can activate mTORC1-KITL signaling in pre-granulosa cells, and these cells trigger the activation of dormant oocytes through KIT-PI3K signaling.^8^ Therefore, the study by Liu *et al.* established an essential communication network between pre-granulosa cells and oocytes. However, it remains unclear as to why only a limited number of primordial follicles are activated at any given time, and how the process is regulated by the surrounding microenvironment.

Ovarian intervention has long been reported to change ovarian development by an unknown mechanism(s). Polycystic ovarian syndrome is thought to occur in 5–10% of reproductively aged women, and surgical treatment with wedge resection or ovarian 'drilling' is the typical choice for anovulation, as opposed to clomiphene or human menopausal gonadotropins.^9^ In women undergoing procedures for bilateral endometriomas, there is a low but definite risk of post-surgical POI, and menopause can then occur earlier than normal.^10^ Comparable studies have reported an immediate decline in antral follicle counts and serum AMH levels after cystectomy, but a partial restoration is normally observed several months later.^11^ Assuming that the surgery will remove both growing and non-growing follicles, the results indicate that the shrinking residual primordial follicle pool will try to re-establish follicular development after the surgery. A recent study found that fragmentation of murine ovaries promoted the development of primary follicles to the late secondary stage by disrupting the Hippo signaling pathway.^12^ However, the effects of such ovarian injury on primordial follicles and the underlying mechanism(s) involved remain unelucidated. We ask whether this represents a common mechanism in selective initial recruitment of primordial follicles.

Herein, by using a murine model with unilateral ovarian surgery, we demonstrated limited activation of primordial follicles near the surgical incision. The dynamic expression of p-rpS6 from somatic cells to primordial follicles then revealed the participation of the somatic mTOR signaling pathway in follicular activation. Furthermore, we found that a localized increase of nerve growth factor (NGF) in the ovarian stroma functioned upstream of mTOR signaling so as to activate primordial follicles after surgery.

## Results

### Activation of primordial follicles after ovarian surgery

**Figure 1:**
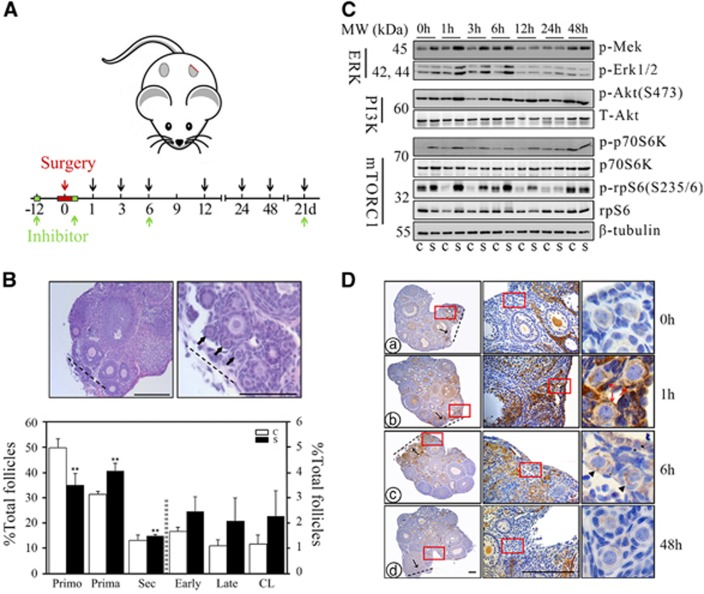
Acceleration of follicular activation and development after ovarian resection. (**A**) Timeline for collecting ovaries after surgery. One-third of one ovary of a pair was resected and the contralateral ovary was remained undamaged as control. Ovaries were collected at 1 h, 3 h, 6 h, 12 h, 24 h, 48 h, or 21 days after surgery. Two injections of inhibitors were administered 12 h before and shortly after the operation, and ovaries were collected at 6 h or 21 days, respectively (green arrows). (**B**) Injury induced an increase in ovarian development. Ovaries were collected 3 weeks after surgery. Upper panel, ovarian histology using hematoxylin and eosin (H&E) staining. Dotted line, original surgical incision. Arrows, clusters of primary follicles. Lower panel, distribution of follicles in paired control (C) and surgically treated (S) ovaries (*n*=5). Data are shown as means±S.E.M. **P<0.01, compared with controls. Primo, primordial follicle; Prima, primary follicle; Sec, secondary follicle; Early, early antral follicle; Late, late antral follicle; CL, corpus luteum. (**C**) Western blots of ovarian proteins using specific antibodies for p-Mek, p-Erk1/2, p-Akt (S473), Akt, p-p70S6k (T389), p70S6k, p-rpS6 (S235/6), rpS6, and *β*-tubulin. *β*-Tubulin was used as a loading control. (**D**) Dynamic changes in p-rpS6 near the incision site at 0 h (a), 1 h (b), 6 h (c) and 48 h (d) after surgery. Middle and right panels, higher magnifications of left panel showing primordial follicles near the incision. Red frames, magnified views were shown in the middle and right panels, respectively. Dotted line, surgical incision. Black arrows, localized expression of p-rpS6 near the incision. Red arrows, pre-granulosa cells. Black arrow heads, primordial oocytes. All bars=100 *μ*m

To observe the effects of surgical intervention on ovarian development, we established a murine model by randomly removing 1/3 of one ovary and leaving the contralateral ovary undamaged (Figure 1A). As primordial follicles immediately enter growth once activated, we first collected paired ovaries 3 weeks later to follow follicular development after surgery. From the ovarian morphology, we clearly identified the incision, and observed clusters of primary follicles or early secondary follicles near the site (Figure 1B, upper panel). Despite the decrease in total follicle numbers, the percentage of follicles at different developmental stages changed markedly in the operated ovary. Compared with the control side, the proportion of primordial follicles decreased, whereas the proportion of growing follicles increased significantly on the surgical side (Figure 1B). Real-time PCR results also detected increased expression of genes related to follicular growth and development (*Cyp17a1*, *Cyp19a1*, *Star*, *Lhr* and *Fshr*) in operated ovaries (Supplementary Figure S1A). Our results indicate that ovarian injury can activate primordial follicles and promote the development and growth of follicles.

To evaluate which signaling pathway has the potential to activate primordial follicles after ovarian surgery, we collected ovaries at different time points and evaluated changes in the PI3K/Akt, mTORC1 and Erk/MAPK pathways using western blot analysis. These three pathways have also been previously reported to be stimulated during tissue damage.^13, 14, 15^ As shown in Figure 1C, the phosphorylation levels of Mek, Erk1/2 in the Erk/MAPK pathway, Akt in the PI3K pathway, and S6K1 and rpS6 in the mTORC1 pathway increased immediately after surgery and peaked approximately 1 h later. Afterward, the higher phosphorylation status was maintained for a time and then decreased gradually. The significant decline in the Erk/MAPK pathway occurred 6 h post-surgery, whereas in the PI3K and mTOR pathways, this occurred 12 h later. The activities of all the signaling pathways were restored to basal levels 24 h later (Supplementary Figure S1B). The stimulation of signaling pathways suggests their involvement in follicular activation after ovarian surgery.

A previous study showed an induction of p-rpS6 in primordial follicles after newborn mouse ovaries were treated with mTOR activators.^16^ Immunostaining for p-rpS6 was then applied to detect the activation of primordial follicles after surgery. Coincident with the immunoblotting results, the expression of p-rpS6 was induced immediately in interstitial cells near the incision site followed by a significant increase thereafter (Figure 1Da-c). We noted that the significant increase in p-rpS6 was only restricted around the incision and showed dynamic changes within primordial follicles. Concomitant with the spread of p-rpS6 in interstitial cells around the wound, p-rpS6 started to be expressed in pre-granulosa cells and oocytes of primordial follicles 1 h after the surgery (Figure 1Db); and 6 h later, stronger signals for p-rpS6 were primarily focused on oocytes in primordial follicles (Figure 1Dc). At 48 h post-surgery, p-rpS6 signals in both primordial follicles and interstitial cells were restored to control levels (Figure 1Dd). p-rpS6-positive primordial follicles in control and injured ovaries were then counted 6 h after surgery, and showed a significant increase in the injured ovaries (~55%) as compared with controls (~15%) (Supplementary Figure S1C). However, no difference was observed 48 h after injury (Supplementary Figure S1C). As the expression of Foxo3a migrates from oocyte nucleus to cytoplasm in activated primordial follicles,^17^ we also used this molecule to label activated primordial follicles in injured ovaries at 6 h post-surgery. As shown in Supplementary Figure S1D, the expression of Foxo3a in oocytes of primordial follicles was translocated from nucleus to cytoplasm near the incision, whereas it maintained its nuclear staining in oocytes a distance from the wound. These results suggested that signals inducing follicular activation after surgery came from ovarian interstitial cells, occurring at about 6 h after surgery.

### Injury-induced follicular activation is blocked by the mTOR inhibitor rapamycin

**Figure 2:**
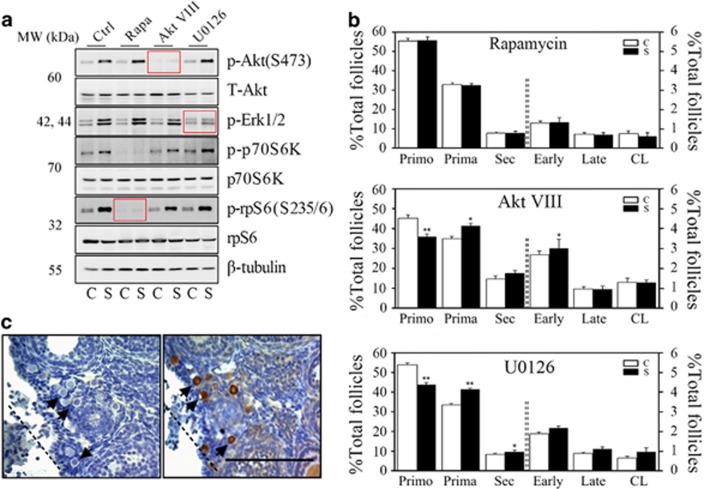
Injury-induced follicular activation is blocked by the mTOR inhibitor rapamycin. Mice were treated with specific inhibitors of the signaling pathways 12 h before surgery and another injection was given just after the surgery. Ovaries were collected at 6 h or 3 weeks after the surgery. C, control ovaries; S, surgically treated ovaries; rapamycin, mTORC1 inhibitor; Akt VIII, Akt inhibitor; U0126, MAPK inhibitor. (**a**) Specific inhibition of corresponding signaling pathways by inhibitors. Ovaries were collected at 6 h for western immunoblotting analysis. The red block represents the phosphorylation level of the corresponding signal marker after treatment with its specific inhibitor. (**b**) Distributions of follicles in paired ovaries (*n*=5) after treatment with inhibitors. Ovaries were collected 3 weeks after surgery. Data are shown as means±S.E.M. **P<0.01, *P<0.05 compared with controls. Primo, primordial follicle; Prima, primary follicle; Sec, secondary follicle; Early, early antral follicle; Late, late antral follicle; CL, corpus luteum. (**c**) Immunostaining of p-rpS6 (left) and Foxo3a (right) near the incision after rapamycin treatment. Injured ovaries were collected 6 h after surgery. Dotted lines, incision; arrows, primordial follicles. Bars=100 *μ*m

Owing to the marked changes in p-rpS6 signals from the stroma to primordial follicles after surgery, we hypothesized that the mTOR signaling pathway participates in the induction of primordial follicles near the incision site. Mice were administered two injections of specific inhibitors that included the mTOR inhibitor rapamycin, the PI3K inhibitor AKT VIII and the MAPK inhibitor U0126 12 h before and shortly after the surgery (Figure 1A). Compared with non-injured-ovary controls, all of the inhibitors showed specific blocking of their corresponding signaling pathways in injured ovaries 6 h after surgery (Figure 2a, red frames). Follicles at different developmental stages were then evaluated in paired ovaries collected 3 weeks later. Only the rapamycin-treated group showed similar follicle proportions between control and injured ovaries (Figure 2b). In Akt VIII- and U0126-treated mice, the acceleration of follicular development was still observed as an increase in the percentage of growing follicles (Figure 2b). Furthermore, only in rapamycin-treated mice, it showed complete inhibition of p-rpS6 immunostaining on the surgically treated ovary, where Foxo3a maintained its nuclear staining in primordial oocytes 6 h after surgery (Figure 2c). These results suggested that stimulation of the mTOR signaling pathway in ovarian stroma was important for injury-induced activation of primordial follicles.

### Post-transcriptional regulation of mTOR signaling pathway after ovarian surgery

**Figure 3:**
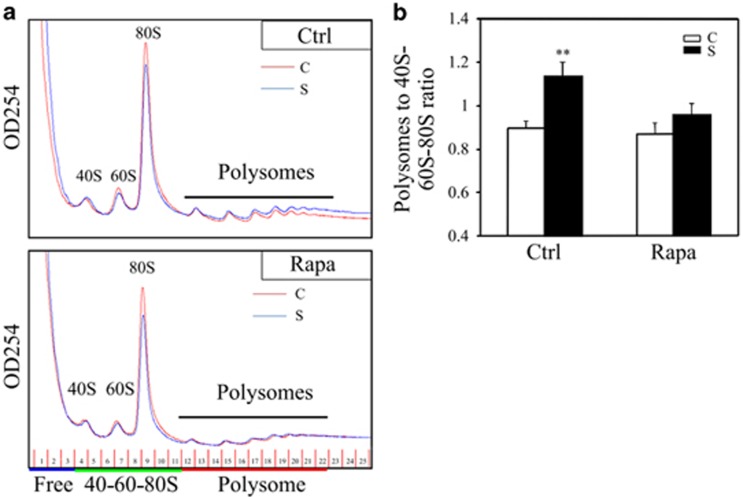
Rapamycin treatment inhibits the increase in global translation after ovarian surgery. Ovaries were collected 6 h after the operation. (**a**) Polysome profiles of control (C) and surgically treated (S) ovaries with or without injections of rapamycin. (**b**) The polysome/40S-60S-80S ratio was quantified by measuring the areas under the polysome and 40S-60S-80S peaks. Three independent experiments were performed. Data are presented as means±SEM; **P<0.01, showing the significant increase in translation by operated ovaries compared with control, non-operated ovaries

**Figure 4:**
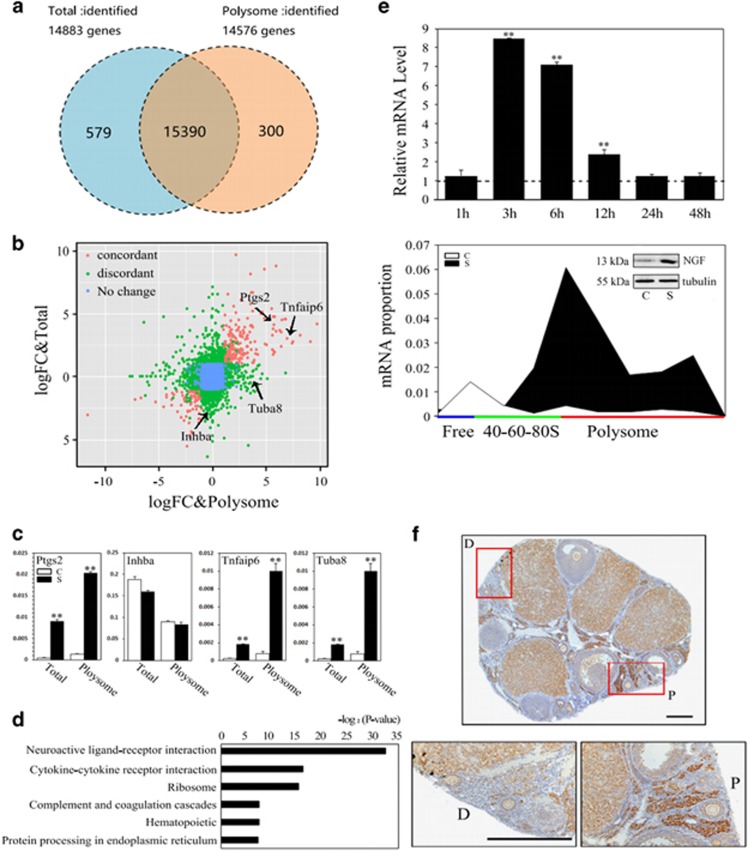
Post-transcriptional regulation of gene expression after ovarian surgery. Paired ovaries were collected at 6 h after surgery for total or polysome RNA-seq. C, control ovaries; S, surgically treated ovaries. (**a**) Venn diagram showing the overlap between genes detected in total and polysome RNA-seq. (**b**) LogFC (fold change) values from total and polysome samples were compared. The logFC values (S *versus* C) for the polysomal fraction (*x* axis) were plotted against the logFC values for the total fraction (*y* axis). The data points are colored according to the change in each fraction. Blue, genes that were not differentially expressed in each fraction; red, genes displaying changes in expression in both sets of conditions had high (or low) values of logFC (>1.0/<−1.0); green, differentially expressed genes either in the polysome fraction or in the total fraction. (**c**) Real-time RT-PCR validation of RNA-seq data randomly selected in each fraction. Data are displayed as means±S.E.M. **P<0.01, compared with control ovaries. (**d**) KEGG analysis of differentially expressed genes of polysome RNA-Seq (FDR<0.001). (**e**) Real-time RT-PCR was performed on the expression of Ngf in total (upper panel) and polysome-associated mRNAs (lower panel). Total mRNAs were collected at 1 h 3, 6 , 12, 24 or 48 h after ovarian surgery. Ovaries were collected at 6 h after surgery for polysome profiling. Fractions represented free RNA (blue, fractions 1–3), 40-60-80S (green, 4–11) and polysome (red, 12–25) were collected for RNA isolation and real-time PCR. Inset (lower panel), western blot of NGF protein expression and the expression of *β*-tubulin was used as internal control. (**f**) Differential expression of NGF in the proximal (P) and distal areas (D) of the incision 6 h after surgery (red frames). Lower, amplifications of P or D areas in red frames. Bar=100 *μ*m

As a major molecular hub that integrates multiple signaling pathways, mTORC1 is a master regulator of protein synthesis that couples nutrient sensing to cell growth and proliferation.^18^ As the mTORC1 inhibitor rapamycin can effectively block surgery-induced follicular activation, we tried to study the mechanism from a translational perspective by polysome profiling. Polysome profiling has been used extensively to investigate cellular translational status under various physiologic conditions and environmental stresses.^19, 20^ As shown in Figure 3a (upper channel), ovarian injury resulted in a global translational increase 6 h after surgery, and the ratio of the polysome to 40S-60S-80S area under the curve showed a 26.8% increase compared with controls (Figure 3b). However, this increase was completely blocked with rapamycin treatment (Figure 3a, lower channel; and Figure 3b). We then isolated the total and polysomal mRNAs for RNA-seq analysis. In both cases, we compared control and the contralateral surgically treated ovaries collected 6 h after surgery. Genes detected by total and polysomal RNA-seq showed a remarkable overlap, indicating that most transcribed mRNAs were translated (Figure 4a and Supplementary Table S1). We then compared differentially expressed protein-coding genes between non-operated and operated ovaries in total and polysome-associated samples (FDR<0.05). Among the 2280 and 1498 genes that were differentially expressed in total and polysomal fractions, respectively, the expression of 596 genes were overlapped; 1684 were differentially expressed in the total sample and 902 were only changed in polysomal fractions (Figure 4b and Supplementary Table S2). The differentially expressed genes were further validated by real-time RT-PCR of randomly selected genes in each fraction (Figure 4c and Supplementary Figure S2A). The results reflected the existence of a post-transcriptional regulation after ovarian surgery. KEGG analysis using differentially expressed genes in polysome samples revealed that neuroactive ligand-receptor interactions were among the top pathways responding to injury (FDR<0.001) (Figure 4d and Supplementary Table S3). By mining neuroactive ligand-receptor interaction-related genes, we found that NGF (but not the other members of the neurotropin (NT) family), increased significantly in operated ovaries in both total and polysomal fractions (Supplementary Figure S2B). RT-PCR of total mRNAs collected at different time points after surgery demonstrated the dynamic expression of NGF, whereas the shift in NGF mRNA to polysomal fractions and the western blotting result showed increased NGF translation 6 h after surgery (Figure 4e). Interestingly, immunohistochemistry of NGF revealed a localized restriction of NGF in interstitial cells near the incision (Figure 4f). The results suggested that localized induction of NGF in interstitial cells may participate in regulating selective follicular activation after surgery.

### NGF functions upstream of mTORC1 in interstitial cells to activate primordial follicles after surgery

**Figure 5:**
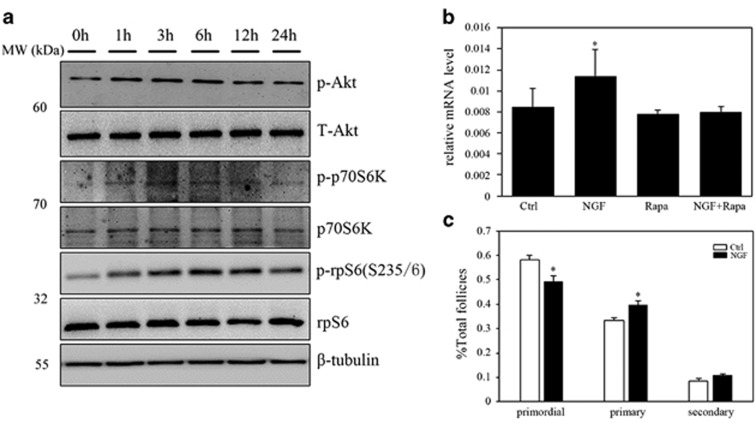
Activation of primordial follicles by NGF through the mTOR signaling pathway. P3 ovaries were treated with NGF, rapamycin or NGF together with rapamycin for 24 h. Twenty-four hours later, they were washed out and the culture was continued for an additional 96 h. (**a**) Stimulation of the mTOR signaling pathway after treatment with NGF. Ovaries were collected at 1, 3, 6, 12 or 24 h. Ovarian proteins were detected by western blots using specific antibodies for PI3K and mTOR signaling pathways. (**b**) The expression of Kitl in ovaries from differently treated groups after 24 h of treatment. The expression of Actb was used as an internal control. (**c**) Follicle counting of ovaries (*n*=5) after treatment with NGF. Ovaries were collected after 96 h of culture *in vitro*. All data shown are from three independent experiments. The data are expressed as means±S.E.M. *P<0.05, as compared with controls. Primo, primordial follicle; Prima, primary follicle. Sec, secondary follicle

**Figure 6:**
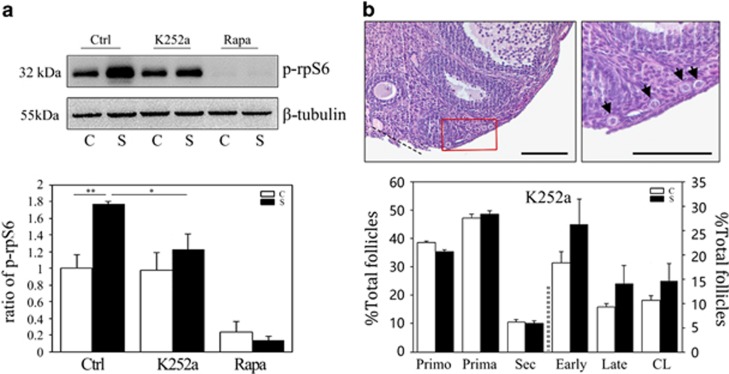
Inhibition of follicular activation by the NGF inhibitor K252a after ovarian surgery. Mice received two injections of K252a before and shortly after surgery. Ovaries were collected at 6 h or 3 weeks, respectively. (**a**) Western blots of ovarian proteins with specific antibodies for p-rpS6(S235/6) and *β*-tubulin. *β*-Tubulin was used as an internal control. Lower panel, densitometry of western blots was quantified and shown by p-rpS6 to *β*-tubulin ratios. The ratios are presented as means±S.E.M. of three independent experiments. *P<0.05; **P<0.01. (**b**) Ovarian morphology and follicle counts 3 weeks after operation. Upper panel, H&E staining of injured ovaries; dotted lines, surgical incision; red frame, primordial follicles near the incision site shown in the right picture (black arrows). All bars=100 *μ*m. Lower panel, distributions of follicles in paired ovaries (*n*=5) after treatment with K252a. No differences were observed between control and surgically treated ovaries. Primo, primordial follicle; Prima, primary follicle; Sec, secondary follicle; Early, early antral follicle; Late, late antral follicle; CL, corpus luteum

To assess whether NGF activates primordial follicles, ovaries from newborn (P2.5) mice were treated with NGF and collected for analysis in the *in vitro* culture system. Western blot analysis showed dynamic changes in the phosphorylation of Akt, p70S6K and rpS6 in the first 24 h of treatment (Figure 5a). By using Kitl (kit ligand) as a marker to evaluate follicular growth after 24 h of culture, we observed that NGF significantly increased *Kitl* mRNAs, and that this increase could be completely blocked with the mTORC1 inhibitor rapamycin (Figure 5b). Follicle counts also showed that NGF treatment accelerated follicular development after 5 days of *in vitro* culture (Figure 5c). Pretreatment with the NGF inhibitor K252a was then applied instead of rapamycin, with two injections at 12 h before and shortly after the surgery. Unlike the complete blocking of p-rpS6 in both control non-operated and operated ovaries, treatment with K252a effectively inhibited the increase in p-rpS6 expression in operated ovaries 6 h after surgery (Figure 6a). Three weeks later, when ovaries were collected to check for follicular development, primordial follicles were still detected near the incision site and the proportions of follicles at different stages manifested no differences between paired controls and surgically treated ovaries (Figure 6b). Our results suggested that surgery induced a localized upregulation of NGF in interstitial cells, which activated the surrounding primordial follicles through the mTORC1 signaling pathway.

### Increased activation of primordial follicles after ovulation

**Figure 7:**
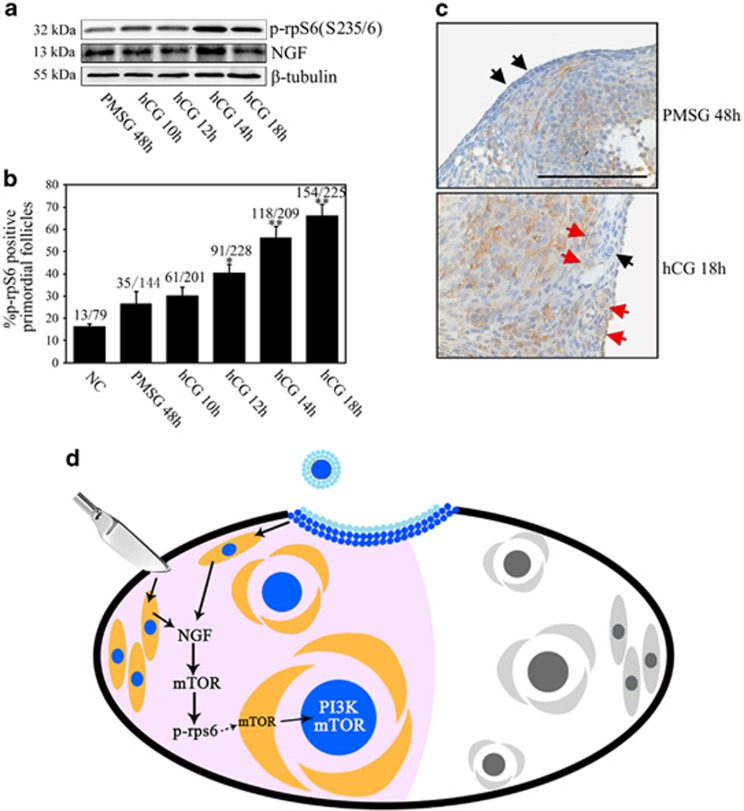
Ovulation-induced primordial follicle activation. Mice at P25 received one i.p. injection of PMSG and followed by another injection of hCG 48 h later. Ovaries were collected at the indicated time points (NC, PMSG 48 h, hCG 10 h, hCG 12 h, hCG 14 h and hCG 18 h). NC, control animals without any hormone injection. (**a**) Expression of NGF and p-rpS6 proteins by immunoblotting. (**b**) Proportions of p-rpS6-positive primordial follicles. Two serial sections from each ovary were used for p-rpS6 staining and at least three ovaries were chosen from each group. *P<0.05; **P<0.01 as compared with NC controls. (**c**) Expression of p-rpS6 in superovulated mouse ovaries 48 h post-PMSG (left panel) and 18 h post-hCG (right panel). Black arrows, p-rpS6-negative primordial follicles; red arrows, p-rpS6-positive primordial follicles. Bar=100 *μ*m. (**d**) Schematic diagram showing selective activation of primordial follicles in mouse ovaries. Ovarian surgery or ovulation-induced injury immediately stimulated localized expression of NGF in interstitial cells (pink area). The stromal mTOR signaling pathway was then activated and this was followed with the sequential activation of the mTOR signaling pathway in pre-granulosa cells and toward oocytes. The primordial follicles were thus finally activated. However, those primordial follicles a distance from the stimuli maintained their dormancy (white area)

In mammalian ovaries, the process of ovulation manifests similarities to minor trauma that occurs naturally and cyclically in the body. We hypothesize that ovulation will also activate primordial follicles through a similar mechanism. Ovaries were collected at different time points (NC, PMSG 48 h, hCG 10, 12, 14 and 18 h, respectively) after superovulatory stimulation. Western blots demonstrated the increase of NGF and p-rpS6 expression after hCG injection and both reached a peak at hCG 14 h (Figure 7a). Follicular activation was then evaluated by p-rpS6 staining and follicle counting results revealed the gradual increase of p-rpS6-positive follicles in ovulated ovaries from hCG 10–18 h. At hCG 18 h, nearly 68% of counted primordial follicles are p-rpS6 positive, which is 4 times and 2.8 times more than that in non-stimulated (NC) and PMSG primed (PMSG 48 h) ovaries, respectively (Figure 7b). Immunostaining also showed the increase of p-rpS6-positive primordial follicles in ovaries collected at hCG 18 h and both p-rpS6-positive and -negative primordial follicles could be detected in the same view (Figure 7c). The results suggest that the ovulation can activate primordial follicles through increase of NGF and the mTOR signaling pathway.

## Discussion

In this study, we demonstrated localized activation of primordial follicles after ovarian trauma. The surgery-induced transient increase in mTORC1 signals in interstitial cells was essential for follicular activation near the incision site. As the translational levels increased markedly in the injured ovary, it was found that the neurotrophic growth factor family member, NGF, participated in activating dormant follicles through the mTOR signaling pathway. We propose that, after ovarian surgery, the rapid increase in NGF levels near the incision site activates the local stromal mTOR signaling pathway, followed by the sequential activation of mTORC1 from pre-granulosa cells to oocytes, and then finally triggers activation of dormant follicles (Figure 7d). This may represent a common mechanism for the selective recruitment of primordial follicles that is determined by signaling changes in the surrounding microenvironment.

It has long been acknowledged that resting primordial follicles are under constant inhibition and remain dormant until activated by the surrounding microenvironment.^6^ A recent study showed that follicular activation is initiated by the upregulation of mTORC1 signals in pre-granulosa cells.^8, 21^ Our study also found progressive activation of primordial follicles in operated ovaries, but the initial signals started in the surrounding interstitial cells. The sequential and limited activation of primordial follicles appears to be reflected by the following aspects: first, after ovarian surgery, although there are many signaling pathways stimulated immediately, only the activation of mTORC1 signals was restricted to the incision, showing dynamic changes in different cell types; second, the two markers of follicular activation, p-rpS6 and Foxo3a, showed differential expression in primordial oocytes in the proximal and distal areas of the incision; third, compared with the other inhibitors, only the pretreatment with the mTOR inhibitor rapamycin blocked the effects on follicular development accelerated by ovarian injury; finally, in rapamycin-treated ovaries, accompanied by the complete inhibition of mTORC1 signals, the inhibition of follicular activation was also reflected in the nuclear location of Foxo3 in oocytes near the incision. As primordial follicle activation is gonadotropin independent, the primordial-to-primary follicle transition is coordinated primarily by locally produced regulatory factors that are produced by pre-granulosa cells of primordial follicles, granulosa cells of growth follicles, and/or stromal cells.^1, 22^ Using the ovarian surgery model, our results show the important role played by the interstitial mTOR signaling pathway with respect to limited and sequential activation of primordial follicles near the incision.

When injury occurs, the wound experiences four distinct but overlapping phases: hemostasis, inflammation, proliferation and remodeling.^23^ As the major intracellular signaling pathways involved in cellular responses in both normal and pathologic conditions, the PI3K/Akt/mTOR and MAPK pathways have been extensively studied following primary mechanical trauma where these cascades are normally associated with cell survival and tissue regeneration.^12, 14, 24^ mTOR, also known as 'mechanistic TOR,' is a large serine/threonine kinase that functions in regulating key cellular responses to energy or nutrient variations within the cell. It exists in two biochemically and functionally distinct complexes, mTORC1 and mTORC2, which differ in their sensitivity to rapamycin. Primary effectors of mTORC1 are p70S6K1 (ribosomal protein p70S6 kinase) and 4E-BP1 (4E binding protein-1). The activation of p70S6K1 phosphorylates its downstream rpS6 (ribosomal protein S6), which initiates a variety of translation-associated activities.^25, 26^ In this study, the elevation of polysome-associated mRNAs denotes a significant increase in global translation after ovarian surgery. This may be one common mechanism that tissues use to respond to trauma and prepare for wound healing.^27^ In the ovary, although there is controversy regarding the existence of female germline stem cells, the long-held view on primordial follicles concerns their non-renewable property.^28, 29^ Therefore, it is certain that primordial follicles will be recruited into the growing pool to replenish the loss caused by ovarian damage. Such accelerated depletion of primordial follicles can also be seen in animals with unilateral ovariectomy or in women in their perimenopausal years.^30, 31, 32^ In this study, only the mTOR inhibitor rapamycin completely blocked the injury-induced activation of primordial follicles. It is possible that the other two pathways participate in regulating other aspects of tissue remodeling as they do in the central nervous system, including neuronal or vascular regeneration.^15, 33, 34^ However, our study revealed that only the mTOR signaling pathway (especially the mTOR signaling pathway in interstitial cells) was specifically involved in regulating primordial follicle activation within the injury penumbra.

The NTs are a family of small polypeptide growth factors that contain 5 members, NGF, brain-derived neurotrophic growth factor, NT-3, NT-4/5 and NT-6. Evidence is mounting that NTs have important roles not only in the peripheral and central nervous systems but also in normal ovarian development and functioning.^35, 36, 37^ In our study, in contrast to the other members of the neurotropins, polysome RNA-seq revealed a marked increase in NGF mRNA after ovarian surgery; and this was further verified by real-time PCR, western blotting and immunohistochemistry. Further studies using *in vitro* cultured newborn mouse ovaries demonstrated that the stimulatory effect of NGF on primordial follicles was mediated by the activation of the mTOR signaling pathway. When an inhibitor of the NGF receptor (TrkA), K252a, was injected into operated mice, it effectively blocked the increase in follicular activation in the operated ovary. NGF binds to its respective receptor TrkA and/or P75NTR to mediate different cellular functions through various downstream signaling pathways, such as PI3K, Erk/MAPK to mediate survival and differentiation, RhoA kinase for cytoskeletal organization and neurite outgrowth, or the JNK pathway for apoptosis through P75NTR only.^38^ Investigators have also reported the involvement of mTOR signals in NGF-mediated survival, cell migration, or VEGF production in different cell types.^39, 40^ As a wound exerts a disruptive effect on the normal anatomical structure of the ovary, healing occurs in a very orderly and efficient manner in order to restore its anatomic continuity and function. It is also possible that NGF participates in regulating other aspects during ovarian remodeling; for example, neural or vascular regeneration through other signaling pathways. However, at least in our study, the involvement of NGF in selective activation of primordial follicles was mediated by the mTOR signaling pathway.

Mammalian ovulation is a distinct biologic phenomenon that requires the rupture of follicles at the surface of the ovary. In the older experimental literature, ovulation was linked to acute inflammation responding to a variety of factors.^41^ Thus, the process of ovulation may be similar to that of minor acute tissue trauma, but occurs naturally and undergoes cyclic phases in the body. In fact, our results showed significant increase of follicular activation after superovulation. As the expression of NGF has shown to be concomitant with the preovulatory rise of gonadotropins in ovaries of many species,^37^ our collective data obtained from ovarian surgery suggested that a similar mechanism exists for ovulation-stimulated follicular activation. Findings from previous studies have suggested a possible role for NGF in many events surrounding ovulation.^34^ In alpacas, for example, *in vivo* treatment with NGF induced ovulation.^42^ Herein, in contrast to the many known functions of NGF in ovulation, we demonstrated a close link between the ovulatory rise in NGF levels and primordial follicle activation. This may represent a new mechanism by which NGF regulates initial follicular recruitment under physiologic conditions via the mTOR signaling pathway (Figure 7d).

In summary, our study showed selective activation of primordial follicles near the incision site after ovarian surgery. This trauma induced a local increase in NGF within interstitial cells, and is involved in regulating follicular activation through the mTOR signaling pathway. The injury accelerated follicular activation and development, and can be used to explain the success of polycystic ovarian syndrome treatment by ovarian wedge resection or ovarian laser or diathermy 'drilling.' Furthermore, although ovarian surgery has been widely applied for removing of ovarian cysts and in order to rescue follicles, it appears that it is much better to preserve primordial follicles so as to avoid the detrimental effects induced by the injury itself. Therefore, blocking the mTOR signaling pathway or NGF function may be a good choice for fertility preservation in women undergoing ovarian surgery.

## Materials and methods

### Experimental animals

CD1 female mice at 6 weeks of age were obtained from Vital River Laboratories (Beijing, China) and housed in the animal facility at Nanjing Medical University. Mice were maintained under a 12/12-h dark–light cycle at 22 °C with free access to food and water. All animal protocols were approved by the Committee on the Ethics of Animal Experiments of Nanjing Medical University. The mice were anesthetized with ketamine hydrochloride (80 mg/kg) and xylazine (16 mg/kg; K113, Sigma, St. Louis, MO, USA). Partial ovarian resection was performed unilaterally with the contralateral ovary left unoperated as a control (Figure 1a). Mice were killed at 1 h, 3 h, 6 h, 12 h, 24 h, 48 h, or and 3 weeks, respectively, and paired ovaries were collected for analysis. In another experiment, inhibitors, including MAPK inhibitor U0126 (2 mg/kg, S1102, Selleckchem, Houston, TX, USA), Akt inhibitor Akt VIII (20 mg/kg, 612847-09-3, MCE, Shanghai, China), mTOR inhibitor rapamycin (2 mg/kg, R8781, Sigma) or NGF inhibitor K252a (500 ug/kg, 2013, Biovision, Milpitas, CA, USA), were separately injected into the mice 12 h before ovarian surgery with another injection performed just after the surgery. The dose for inhibitor injections were used according to previous reports.^43, 44, 45, 46, 47^ Paired ovaries were collected at 6 h or 3 weeks for further analysis. To evaluate follicular activation after superovulation, CD1 mice at 25 days of age received one i.p. injection of PMSG (5 IU, 110253130, Ningbo, China) followed by another injection of hCG (5 IU, 110251282, Ningbo) 48 h later. Ovaries were collected 48 h post-PMSG and 10 h, 12 h, 14 h or 18 h post-hCG for western blot analysis and immunohistochemsitry.

### Follicle counting

Ovaries from operated mice, superovulated mice, and *in vitro* cultured ovaries from newborn mice were collected and fixed in 10% buffered formalin for 12 h, embedded in paraffin, serially sectioned at a thickness of 5 *μ*m, and then stained with hematoxylin and eosin. To evaluate follicular development in operated mice, all follicles were counted at every fifth section using the fractionator and nucleator principles.^16^ Follicles were only counted when the dark-staining nucleolus was seen within the nucleus of the oocytes to prevent recounting the same follicle. To evaluate the activation of primordial follicles, two serial sections from the largest cross-section through the center of each ovary were chosen for p-rpS6 staining. The average of p-rpS6-positive primordial follicles/total primordial follicles was used as the percentage of activated primordial follicles in each section. We used the same standards to count follicles in *in vitro* cultured newborn mouse ovaries. All sections were counted by two independent individuals for comparison.

### Immunohistochemistry

Paired ovaries were collected at 6 h after ovarian resection to detect the expression of p-rpS6, Foxo3a and NGF. Briefly, 5 *μ*m sections were deparaffinized, rehydrated and endogenous peroxidase activity was blocked by incubation in 3% hydrogen peroxide in methanol for 20 min. Antigen retrieval pretreatment was carried out by boiling the sections in 0.01 M citrate buffer, pH 6.0 for 10 min. Immunohistochemical analyses were performed using a Histostain Kit (856743, Invitrogen, Carlsbad, CA, USA) with antibodies against p-rpS6 (S235/236) (4858, Cell Signaling Technology, Beverly, MA, USA), Foxo3a (ab53287, Abcam, Cambridge, MA, USA) and NGF (ab6199, Abcam) overnight at 4 °C. For some sections, primary antibodies were replaced with non-immune rabbit IgG as negative controls.

### Immunoblotting analyses

Proteins were extracted by RIPA lysis buffer (P0013B, Beyotime Institute of Biotechnology, Beijing, China) with protease inhibitor cocktails (M221, Amresco, Solon, OH, USA). Proteins were separated by electrophoresis and after electronic transfer, the membranes were blocked and incubated with specific antibodies overnight at 4 °C. Antibodies against S6K1 (2708), p-S6K1 (T389) (9234), rpS6 (2217), p-rpS6 (S235/6), p-Erk1/2 (9101), p-MEK (9121), p-Akt (S473) (9271), Akt (9272) and *β*-tubulin (2128) were all rabbit antibodies and purchased from Cell Signaling Technology. Horseradish peroxidase-conjugated goat anti-rabbit IgG (SC2004, Santa Cruz Biotechnology, Dallas, TX, USA) were then used to detect proteins through enhanced chemiluminescence (RPN2232, Amersham, Washington, NY, USA).

### Real-time PCR analyses

cDNAs were synthesized from total RNAs or polysome-associated RNAs by using a FastQuant RT Kit (TIANGEN Biotech, Beijing, China). SYBR-based qPCR was then performed using Bestar qPCR Mastermix (DBI-2225, DBI Bioscience, Ludwigshafen, Germany) on an ABI StepOnePlus platform (Thermo Fisher Scientific, Waltham, MA, USA). Quantification of various mRNAs was performed by using the actin amplification signal as the internal control. Primer sequences used in the qPCR are listed in Supplementary Tables S4 and S5. The specificity of the PCR products was assessed by melting curve analyses and amplicon size was determined by electrophoresis in 2% agarose gels.

### *In vitro* ovarian organ culture

Ovaries from the P3 female pups were harvested and cultured on inserts (PICM01250, Millipore, Billerica, MA, USA) with 0.4 ml culture medium added to the bottom of each well. Each insert held 4–5 ovaries and a drop of medium was added to the top of each tissue fragment to prevent drying.^17^ The culture medium was MEMalpha supplemented with 0.23 mM pyruvic acid, 50 mg/l streptomycin sulfate, 75 mg/l penicillin G and 3 mg/ml BSA. Ovaries were randomly distributed to the control and the treated groups. In the treated groups, the ovaries were incubated with 250 nM rapamycin (Sigma), 100 nM recombinant mouse beta-NGF (1156-NG/CF, R&D, Minneapolis, MN, USA) or rapamycin together with NGF for 24 h. After washing out the chemicals, the culture continued in control medium for 96 h (24+96 h) and ovaries were collected at 24 and 96 h, respectively.

### Polysome profiling

Control non-operated and operated ovaries were collected at 6 h after surgery. Approximately 20 ovaries were lysed with lysis buffer (100 mM KCl, 0.1% Triton X-100, 50 mM HEPES, pH 7.4, 2 mM MgCl_2_, 10% glycerol, 1 mM DTT, 100 mg/ml cycloheximide, 20 U/ml RNase inhibitor (3335402001, Roche), plus protease inhibitor cocktail (04693116001, Roche, Basel, Switzerland)). Lysates were centrifuged at 12 000* g* for 10 min at 4 °C. Supernatants were divided into two parts, one part for total RNA preparation, whereas the other part was loaded onto a 10 ml 20–50% (W/V) linear sucrose gradient made by Gradient Master (BioComp, Fredericton, NB, Canada) and centrifuged in a SW-41 Ti rotor (Beckman, Brea, CA, USA) for 150 min at 38 000 r.p.m. at 4 °C. Polysome profiles were collected with a fraction collector (Gilson, Middleton, WI, USA) and determined by monitoring RNA absorbance (Bio-Rad, Hercules, CA, USA) at 254 nm. Twenty-five fractions (with each fraction containing 0.45 ml gradients) were collected from the top of the gradients into cold microfuge tubes and immediately placed on dry ice. Fractions 1–11 were designated as non-translated with fractions and fractions 12–25 were designated as polysomes. Gradient fractions were supplemented with Cas9 mRNA to control for the efficiency of RNA recovery.^48^ Total RNA and polysome-associated RNA were precipitated with ethanol overnight and purified with RNeasy Plus Micro kit (74034, Qiagen, Germantown, MD, USA).

### RNA-seq

Total mRNA or polysome-associated mRNA (*μ*g) was subjected to construction of cDNA libraries by using NEB Next Ultra Directional RNA Library Prep Kit for Illumina (E7645-S, NEB, Ipswich, MA, USA). Finished libraries were sequenced by Illumina HiSeq 2000 for 100-bp paired-end sequencing. The RNA-seq reads were analyzed by TopHat,^49^ Cufflinks^50^ and DESeq.^51^ Genes mapped with at least 10 reads were considered as the threshold of quantifiable genes in the RNA-seq data. Multiple testing corrections were used according to the Benjamin Hochberg FDR method. To identify DEGs, stably expressed protein-coding genes were filtered according to statistical significance (FDR<0.05) and greater than twofold changes were considered to be significant. A gene enrichment analysis was performed with DAVID.^52^

### Statistical analyses

The chi-square test, or one-way ANOVA and Mann–Whitney *U*-test were used to evaluate differences between groups. Data are means±S.E.M. A value of *P*<0.05 was considered to be statistically significant.

### Data availability

Transcriptome and translatome sequence reads have been deposited in the NCBI.SRA database with the accession number PRJNA357978. Raw supporting data are provided in the additional files (Supplementary Tables S1).
