# Supplementary material for: Restricting the induction of NGF in ovarian stroma engenders selective follicular activation through the mTOR signaling pathway
Source: Cell Death Dis. 2017 May 25;8(5):e2817–. doi: 10.1038/cddis.2017.168 (PMC5520698; doi:10.1038/cddis.2017.168)
Supplement: Supplementary Figures, Figure Legends and Tables [file cddis2017168x1.docx]

**Supplementary Figures, Figure Legends and Tables**

**
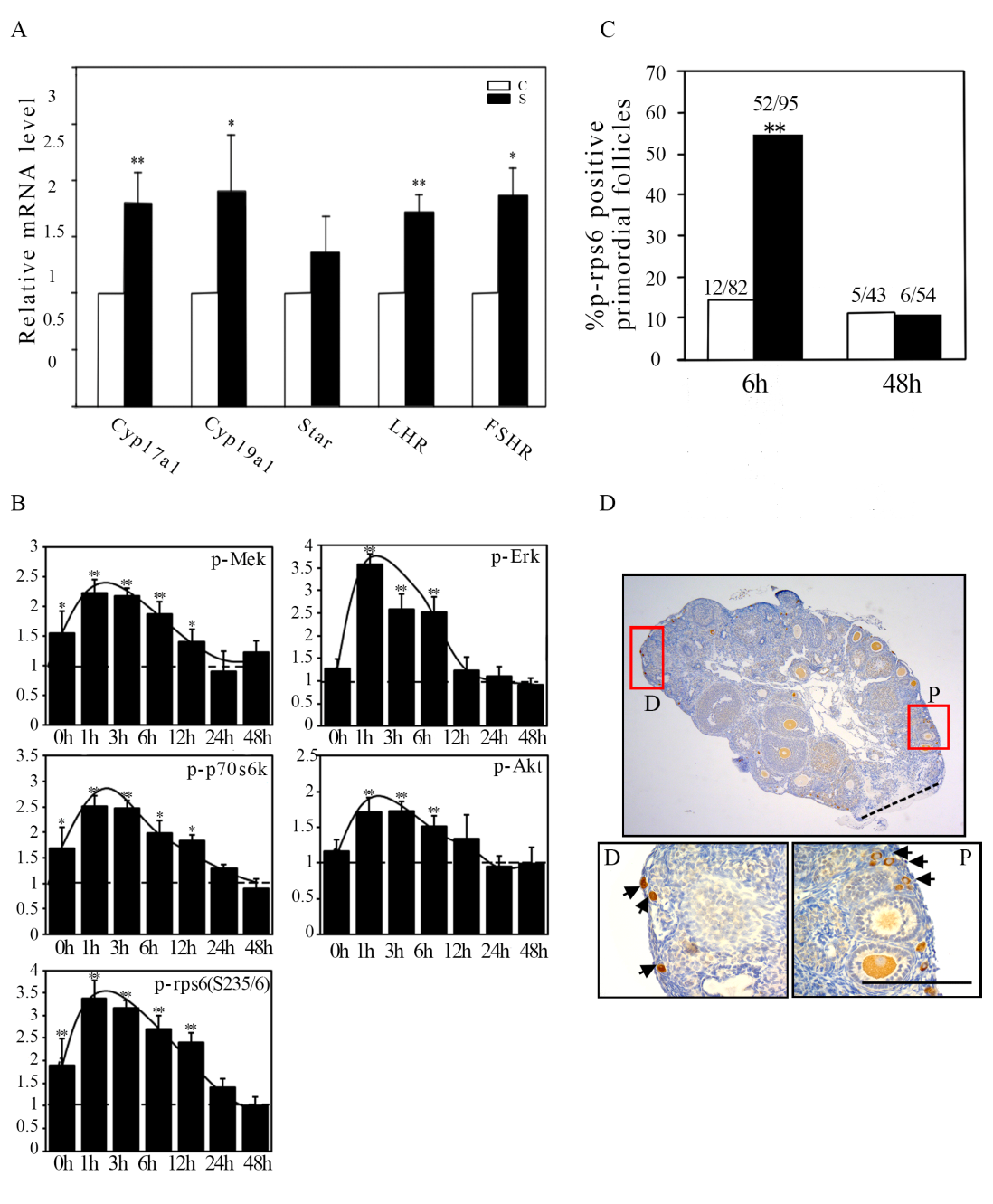
**

**Figure S1.** Activation of primordial follicles induced by ovarian surgery. Paired control, non-operated and operated ovaries were collected at 6 h and 3 w after surgery. A) Real-time PCR of growth-related genes in controls and operated ovaries 3 w after the operation. The data are presented as means±SEM of at least 3 replicates. The expression of *Actb* was used as an internal control and each gene was set to 1 for the control side. B) Densitometry of Western blots (Fig. 1C) was quantified and designated as ratios of phosphorylated proteins to total protein or β-tubulin at each time point. The ratios of corresponding proteins in control non-operated ovaries were set as 1. C) Quantification of p-rpS6-positive primordial follicles (Fig. 1D) in control and injured ovaries 6 h or 48 h after the operation. Two serial sections from each ovary were used for p-rpS6 staining, and at least 3 ovaries were rchosen from each group. D) Immunostaining of Foxo3a in injured ovary after 6 h of surgery. P, proximal to the incision; D, distal to the incision. Lower panel, higher magnifications of red frames represent the differential expression of Foxo3a in region D and region P. All bars=100μm. *P<0.05; **P<0.01, compared to controls.


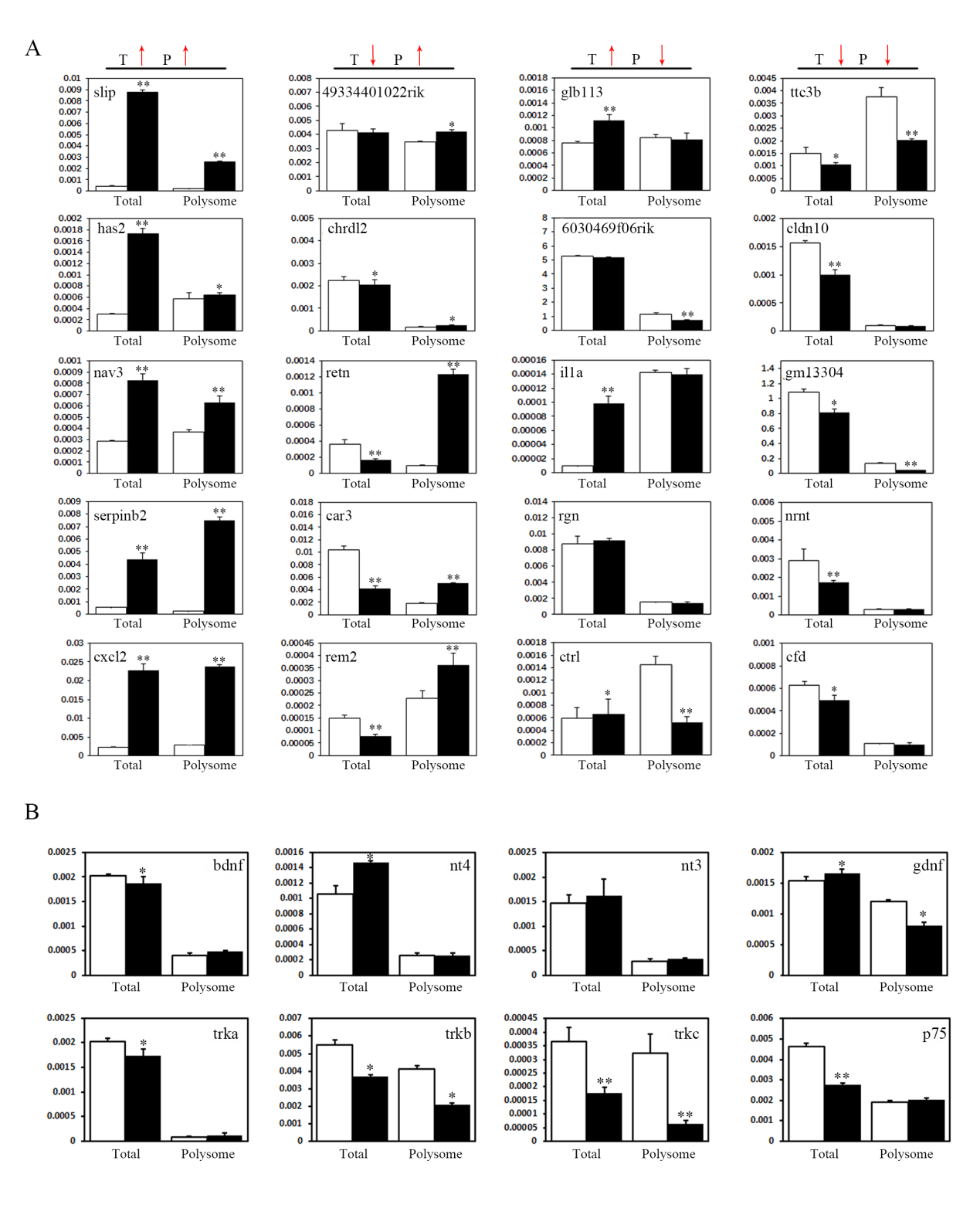


**Figure S2.** Real-time PCR confirming the differential expression of genes in total and polysome- associated RNA-seq (A). Total mRNA and polysome-associated mRNAs were collected 6 h after surgery. T, total mRNAs; P, polysome-associated mRNAs. C, control ovaries; S, surgically treated ovaries. Upward arrow, upregulation; Downward arrow, downregulation. B) Expression of members of the neurotrophic factor family. *P<0.05; **P<0.01 as compared to controls.

**Table S4 Primers of follicular development related genes**

| *Cyp17a1* | GCCCAAGTCAAAGACACCTAAT | GTACCCAGGCGAAGAGAATAGA |
| --- | --- | --- |
| *Cyp19a1* | ATGTTCTTGGAAATGCTGAACCC | AGGACCTGGTATTGAAGACGAG |
| *Star* | TGCCCATCATTTCATTCATCCTT | AAAAGCGGTTTCTCACTCTCC |
| *Lhr* | AGCCATCTCGGTATTCAGGG | GGATTACAGACGCGGAATCATT |
| *Fshr* | CCTTGCTCCTGGTCTCCTTG | CTCGGTCACCTTGCTATCTTG |
| *Kitl* | GAATCTCCGAAGAGGCCAGAA | GCTGCAACAGGGGGTAACAT |
| *Actb* | CCCTAAGGCCAACCGTGAAA | AGCCTGGATGGCTACGTACA |

**Table S5 Primers for verification of total and polysome RNA-seq data**

| **Gene** | **Forward primer** | **Reverse primer** |
| --- | --- | --- |
| *Ngf* | TGTGCCTCAAGCCAGTGAAAT | ACCTTTATTGGGCCCAGACAC |
| *Bdnf* | TTACCTGGATGCCGCAAACAT | TGACCCACTCGCTAATACTGTC |
| *Gdnf* | CCAGTGACTCCAATATGCCTG | CTCTGCGACCTTTCCCTCTG |
| *Trka* | GCCTAACCATCGTGAAGAGTG | CCAACGCATTGGAGGACAGAT |
| *Trkb* | CTGGGGCTTATGCCTGCTG | AGGCTCAGTACACCAAATCCTA |
| *Trkc* | CTGAGTGCTACAATCTAAGCCC | CACACCCCATAGAACTTGACAAT |
| *P75* | CTAGGGGTGTCCTTTGGAGGT | CAGGGTTCACACACGGTCT |
| *Nt3* | GGAGTTTGCCGGAAGACTCTC | GGGTGCTCTGGTAATTTTCCTTA |
| *Nt4* | TGAGCTGGCAGTATGCGAC | CAGCGCGTCTCGAAGAAGT |
| *Slpi* | GGCCTTTTACCTTTCACGGTG | TACGGCATTGTGGCTTCTCAA |
| *Has2* | TGTGAGAGGTTTCTATGTGTCCT | ACCGTACAGTCCAAATGAGAAGT |
| *Nav3* | GAACTCCAGCTACAGCTAGGC | CCTGCATCTGAAGGAGACCTTT |
| *Serpinb2* | GTGCTGGGGGTAACACTGAAC | GCGAAATCACAGCCACTGAAG |
| *Cxcl2* | CCAACCACCAGGCTACAGG | GCGTCACACTCAAGCTCTG |
| *4933440N22Rik* | TCCCGAAAAGTGATCCCAAAG | AACTTCAGACAAATCCCTCCG |
| *Chrdl2* | TTTGCTGGGACTCGTGATGTT | GTGGTTCCAAGTAGGGGTGC |
| *Car3* | TGACAGGTCTATGCTGAGGGG | CAGCGTATTTTACTCCGTCCAC |
| *Retn* | AAGAACCTTTCATTTCCCCTCCT | GTCCAGCAATTTAAGCCAATGTT |
| *Rem2* | ATGCCCGTGCCCTACAAAC | ACGCCATCTTTTTGGGTAAGG |
| *Ttc36* | TCTTCAACCCTGATACCCCCT | GGAGTGCTCCAACTGTGCT |
| *Cldn10* | CGAATGGAAAGTGACCACCC | ATTAGTCCTCTACATGCCTGGAT |
| *Cfd* | CATGCTCGGCCCTACATGG | CACAGAGTCGTCATCCGTCAC |
| *Gm13304* | AACCAGTTCAACCCCAGAAG | CGACTTCCCATTGCTTTTCTG |
| *Nrtn* | GGGCTACACGTCGGATGAG | CCAGGTCGTAGATGCGGATG |
| *6030469F06Rik* | GAGATTGTCAGGAGTTGGAGTC | CCCTGTATCTATTCTGTCCAAGC |
| *Glb1l3* | TGGAAGGACCGCTTGTTGAAG | CCACAGCCCAATAGTCTTAGCA |
| *Rgn* | TCCAAGCCAGACGACGATATT | CACACTCTCCGCTCTTTATCTTC |
| *Ctrl* | AGCCTAACCCTTAGCCTGGTC | TCCCCGTTGACAATTCTCTGA |
| *Il1a* | CGAAGACTACAGTTCTGCCATT | GACGTTTCAGAGGTTCTCAGAG |
| *Tuba8* | GGCCACAGGATGACACACG | ATTCTCCCCAGGGGTATTTGT |
| *Ptgs2* | TGAGCAACTATTCCAAACCAGC | GCACGTAGTCTTCGATCACTATC |
| *Tnfaip6* | GGGATTCAAGAACGGGATCTTT | TCAAATTCACATACGGCCTTGG |
| *Inhba* | TGAGAGGATTTCTGTTGGCAAG | TGACATCGGGTCTCTTCTTCA |
